# Pessimism and the risk for coronary heart disease among middle-aged and older Finnish men and women: a ten-year follow-up study

**DOI:** 10.1186/s12872-015-0097-y

**Published:** 2015-10-02

**Authors:** Mikko T. Pänkäläinen, Tuomas V. Kerola, Jukka J. Hintikka

**Affiliations:** Department of Psychiatry, Päijät-Häme Central Hospital, Keskussairaalankatu 7, FI-15850 Lahti, Finland; Department of Internal Medicine, Päijät-Häme Central Hospital, Lahti, Finland; School of Medicine, University of Tampere, Tampere, Finland

**Keywords:** Pessimism, Optimism, Life Orientation Test - Revised, Coronary heart disease, Gender difference

## Abstract

**Background:**

Despite the growth in knowledge about coronary heart disease (CHD) risk factors, and the advances made in preventing and treating them, the incidence of CHD is still notably quite high. Research has concentrated on the physiological factors that present risks for CHD, but there is an increasing amount of evidence for the connection of mental health, personal traits and CHD. Data on the connection of disposition (optimism or pessimism) and CHD are relatively scarce. The aim of this study was to investigate the long-term connection between optimism, pessimism and the risk for having CHD.

**Methods:**

This was a ten-year prospective cohort study on a regional sample of three cohorts aged 52–56, 62–66 and 72–76 years at baseline (*N* = 2815). The study groups were personally interviewed four times (in 2002, 2005, 2008 and 2012). The revised Life Orientation Test (LOT-R) was completed at the first appointment to determine the level of dispositional optimism or pessimism. During the ten-year follow-up, the incidence of new cases of coronary heart diseases was measured. The association between dispositional optimism/pessimism and the incidence of CHD during the follow-up was studied with logistic regression.

**Results:**

Those who developed coronary heart disease during the ten-year follow-up were significantly more pessimistic at baseline than the other subjects. Using multivariate logistic regression models separately for men and women, we noticed no elevated risk for CHD in the pessimistic women compared to the non-pessimistic women. However, among men in the highest quartile of pessimism, the risk for CHD was approximately four-fold (OR 4.11, 95 % CI 1.68–11.04) that of the men in the lowest quartile. Optimism did not seem to have any role in the risk for developing CHD.

**Discussion:**

Our main finding is that pessimism seemed to be a clear risk factor for coronary heart disease in men even after adjusting for classical well-known risk factors while optimism did not seem to be a protective factor. Connection between pessimism and coronary heart disease was not detectable among women. Similar gender differences between psychosocial factors and overall well-being have been noticed in some earlier studies, too. The mechanism of this gender difference is not fully understood. Differences between men and women in somatic responses to stress found in earlier studies may at least partly explain this phenomenon.

The impact of optimism and pessimism on cardiovascular disease has been studied earlier and several possible mechanisms have been discovered but it seems clear that they cannot fully explain the association. For example, optimists have healthier lifestyles which lowers the risk for coronary heart disease, but pessimism was established to be a risk factor for cardiovascular disease in our study even in logistic regressions including the best known classical risk factors, e.g. smoking and high level of blood glucose. According to our study it is important to pay attention also to the psychosocial components in addition to the well-known risk factors when planning the prevention of coronary heart disease. Measuring pessimism is quite easy and it consumes very little time. Once the amount of pessimism is ascertained, it is easier to define who is in the greatest need of preventive actions concerning coronary heart disease.

**Conclusions:**

Pessimism seems to be a substantial risk factor for CHD, and as an easily measured variable it might be a very useful tool together with the well-known physiological risk factors to determine the risk for developing CHD, at least among men.

## Background

Cardiovascular events are the leading cause of mortality in industrialized countries [1]. There is a significant number of known risk factors for coronary heart disease (CHD). In a study of more than 120,000 subjects, the majority of those with CHD (75 % of women and 80 % of men) had at least one of the four most important physiological risk factors (diabetes, hypertension, smoking or elevated lipids) [2]. There were also patients with no recognized risk factors and subjects with one or more risk factors that still had no CHD. One reason for this might be psychosocial factors that can be either protective or risk factors for CHD.

The terms optimism and its antonym pessimism derive from Latin words ‘optimus’ and ‘pessimus’, respectively, the first meaning ‘the best’ and the latter meaning ‘the worst’ [3]. Optimists have ‘a feeling or belief that good things will happen in the future’, whereas pessimists have ‘the feeling that bad things are more likely to happen than good things’ [4]. People are often categorized as optimists or pessimists. This can lead to the conclusion that optimism and pessimism are the two extremities of the same unidimensional continuum (dispositional optimism). Nevertheless, the concept of optimism itself has long been controversial: there is debate over whether the optimism construct should be seen as one bipolar dimension or if optimism and pessimism should be seen as two separate dimensions that exist simultaneously and may be unattached to each other. Like other personality traits also optimism and pessimism characterize an individual in ordinary situations and they are stable and predictable once they have evolved. Unlike, e.g. mood, they seem to remain the same over situations as well as time, regardless of negative or positive incidents [5–7]. The development of optimism and pessimism appears to be influenced by both heritage and environment [8, 9].

Research on CHD has mainly been focused on physiological risk factors, so the possible psychosocial risk factors are not as well known. Psychosocial factors have nevertheless been considered in some studies. For example, in the INTERHEART study the psychosocial factors were one of the most significant risk factors for myocardial infarction [10, 11]. In most of the studies where psychosocial risk factors have been linked to CHD, the focus has mostly been on psychiatric symptoms and illnesses and not on the role of the construction of personality. Nevertheless, in some studies there has been a connection also between the personality traits, and psychiatric morbidity [12, 13], physical functioning [12, 14], overall mortality [15] and illness burden [16].

The link between physical health and optimism and pessimism as personality traits has not been widely studied. Two long-term follow-up studies on older men have suggested that an optimistic explanatory style protects against CHD [17] and unidimensionally assessed dispositional optimism protects against cardiovascular death [18]. In an extensive study on women, a lower risk for CHD was found among optimists [19]. Optimism was assessed unidimensionally i.e. the optimism and the pessimism were studied as the opposite ends of the same continuum rather than two independent variables. In two follow-up studies on both men and women, optimism reduced the risk for CHD independently of other risk factors [20, 21]. Both studies, however, have methodological shortcomings. To assess optimism, Hansen et al. [20] used only two of the six questions of the revised Life Orientation Test [22] and Boehm et al. [21] used only one self-worded question.

We found no prospective studies on general population samples where the risk for coronary heart disease was evaluated separately for optimism and pessimism in men and women. For this reason, we conducted this ten-year follow-up study on middle-aged and older Finnish men and women. We assessed whether optimism and pessimism are separately genuine protective factors or risk factors for coronary heart disease.

## Methods

A stratified (age, sex, municipality) random sample of men and women born in 1926–30, 1936–40, and 1946–50 were drawn in 2002 from the population registry of all 14 municipalities of the Päijät-Häme region, Finland. A total of 4,272 subjects were invited, and 2,815 (66 %) participated.

In 2002, the study subjects were invited to the launch of the Good Ageing in Lahti region (GOAL) study. The GOAL study primarily aimed to improve the health and well-being of the ageing population of the Päijät-Häme region. The test subjects completed several questionnaires concerning their current life status (e.g. psychosocial background, socio-economic status, health and lifestyle). The use of medications was also documented. Blood tests were taken to specify the levels of blood glucose and cholesterol. Study subjects were measured for height and weight, and their body mass indexes (BMI) were calculated. Waist circumference was measured at a level midway between the lowest rib and the iliac crest. At the launch, the blood pressure of the study subjects was measured three times and the average was documented.

Smoking habits were asked and the study subjects were divided into daily smokers (i.e. persons who smoked every day regardless of the amount) and non-daily smokers. Study subjects who used five or more units of alcohol in one sitting formed the ‘heavy drinkers’ group, while the rest were ‘non-heavy drinkers’. The subgroup ‘regular physical exercise’ included those who exercised for 30 min at least twice a week. Finally, the use of drugs was asked and the following variables were created: use of statins (yes vs no), use of drugs for hypertension (yes vs no) and use of drugs for diabetes (yes vs no).

Optimism and pessimism was measured by using the revised version of the Life Orientation Test (LOT-R). The test was initially developed in the mid-1980s to assess the beneficial effects of optimism on psychological and physiological health (LOT, [23]). The scale was re-evaluated and revised (LOT-R) later by Scheier, Carver and Bridges [22] to focus its item content more closely on the subject’s expectations of the future. Originally, both LOT and LOT-R were thought to be unidimensional scales, but later studies have suggested that they may have two separate independent dimensions, namely optimism and pessimism. Even if in some studies the unidimensional bipolar model of dispositional optimism has been at least as accurate as the bidimensional one [23–25], the separation of optimism and pessimism has been recognized to be very useful in many other studies, leading to the better prediction of outcomes [26–32]. In the bipolar model, optimism and pessimism seem to hide some of each other’s results, and some data may be lost in the process. As a compromise, it has also been suggested that dispositional optimism might be a unidimensional continuum, while the tests used to measure this variable – including the LOT and LOT-R – give answers in two separable dimensions, i.e. optimism and pessimism [33].

LOT-R includes four fillers and six actual statements, of which three are worded positively for optimism (e.g. ‘In uncertain times, I usually expect the best’) and three are worded negatively to indicate pessimism (e.g. ‘If something can go wrong for me, it will’). The respondents are asked to indicate how much they agree with the statements in general, as expressed on a scale from 1 (‘I disagree a lot’) to 5 (‘I agree a lot’). A higher score refers to greater optimism or greater pessimism depending on the statement. In the final analyses, we used the independent optimism component subscale scores and the pessimism component subscale scores separately. They were named ‘optimism’ and ‘pessimism’, respectively.

In 2012, ten years after the GOAL study launch, 1697 subjects responded. They were asked whether they had coronary heart disease that had been diagnosed by a doctor. The study group consisted of those who answered ‘yes’ and the control group of those who answered ‘no’. Those who in 2002 had not participated, had answered incompletely, or reported having coronary heart disease were excluded from the study (*n* = 261). Those who in 2002 reported having coronary heart disease and were excluded from the final sample were more pessimistic than the other participants (pessimism subscale score mean (SD): 4.45 (2.64) vs 3.88 (2.70), *p* = 0.001); for optimism there was no difference (data not shown). The final study group (*n* = 101) consisted of 57 men and 44 women, and the final control group (*n* = 1335) consisted of 580 men and 755 women. Among those who finished the study, the total incidence of coronary heart disease during the ten-year follow up was 8.9 % (57/637) in men and 5.5 % (44/799) in women.

The study protocol was approved (R12013) by the Regional Ethics Committee of Tampere University Hospital. All participants gave their informed consent prior to data collection.

In the statistical analyses, we used exploratory factor analysis with varimax rotation to assess the dimensions of the LOT-R scale. When handling categorical variables we used the Chi-squared test. For continuous variables, we used the nonparametric Mann–Whitney *U* test. Finally, we calculated logistic regression models to discover the fully adjusted odd ratios for those risk factors for CHD that associated (*p* < 0.10) with coronary heart disease in the univariate analyses.

### Availability of supporting data

The data of this study is a part of the GOAL (Good Ageing in Lahti Region) Project. The original data was collected and is preserved by the Palmenia Centre for Continuing Education in Lahti, Finland.

## Results

First, we sought to determine whether optimism and pessimism fall on a unipolar continuum or if they are two different and independent factors. All the questions (without the fillers) of the LOT-R and their answers were included in a factor analysis with varimax rotation and Kaiser normalization. The final solution is shown in Table 1. A clear-cut two-factor solution was found, which strongly suggests that when optimism and pessimism are assessed with the LOT-R scale, they are two separate variables. We decided to handle them separately in further analyses.

**Table 1:** The two-factor structure of the revised Life Orientation Scale in principal component analysis with varimax rotation and Kaiser normalization

	Optimism	Pessimism
In uncertain times, I usually expect the best.	0.717	−0.129
If something can go wrong for me, it will.	0.060	0.778
I am always optimistic about the future.	0.764	0.081
I hardly ever expect things to go my way.	−0.004	0.838
I rarely count on good things happening to me.	0.112	0.812
Overall, I expect more good things to happen to me than bad.	0.667	0.247

There were no differences between men and women in optimism (LOT-R subscale score mean (SD): 8.35 (2.10) vs 8.40 (2.12), Mann–Whitney *U* test *p* = 0.81) or pessimism (LOT-R subscale score mean (SD): 3.60 (2.67) vs 3.55 (2.59), *p* = 0.93). No differences were found in optimism between age groups (ages 52–56 vs 62–66 vs 72–76 years: 8.34 (2.13) vs 8.40 (2.07) vs 8.43, Kruskal-Wallis test *p* = 0.84). Those with a higher age were more pessimistic (3.20 (2.62) vs 3.71 (2.50) vs 4.28 (2.76), *p* < 0.001).

Those who developed coronary heart disease during the ten-year follow-up had been significantly more pessimistic at baseline than the subjects of the control group (LOT-R subscale score mean (SD): 4.43 (2.70) vs 3.51 (2.61), Mann–Whitney *U* test *p* = 0.001). In terms of optimism, there was no difference (LOT-R subscale score mean (SD): 8.49 (1.94) vs 8.37 (2.12), *p* = 0.61). When studied by gender, differences in pessimism scores were the same in the total sample both in men (4.39 (2.73) vs 3.52 (2.66)) and women (4.48 (2.70) vs 3.49 (2.58)), and they were no more statistically significant (*p* = 0.15 and *p* = 0.17, respectively). The LOT-R optimism subscale scores were similar among those who got ill and those who remained healthy both in men (8.56 (1.80) vs 8.32 (2.13), *p* = 0.43) and in women (8.39 (2.12) vs 8.40 (2.12), *p* = 0.93).

Those men who on follow-up reported having CHD had had higher blood glucose levels and higher waist circumferences at baseline than the other men. There was also a trend towards higher BMI and higher systolic blood pressure. The findings in women were the same in both groups, except there was a statistically significant difference in BMI (Table 2).

**Table 2:** Risk factors at baseline and incidence of coronary heart disease in the ten-year follow-up

	Men					Women				
	Coronary heart disease				Mann–Whitney *U* test	Coronary heart disease				Mann–Whitney *U* test
	Yes		No			Yes		No		
	*N* = 57		*N* = 580			*N* = 44		*N* = 755		
	Mean	SD	Mean	SD	*p*-value	Mean	SD	Mean	SD	*p*-value
Body mass index (kg/m^2^)	28.11	3.72	27.26	3.68	0.075	29.11	4.48	27.53	4.85	0.011
Blood glucose (mmol/L)	6.17	1.26	5.71	0.90	0.023	5.97	1.66	5.34	0.79	<0.001
Cholesterol (mmol/L)	5.94	0.97	5.76	1.05	0.123	5.98	1.18	5.94	0.99	0.828
Waist circumference (cm)	100.9	9.2	98.4	10.5	0.044	94.4	12.4	90.0	12.7	0.017
Systolic blood pressure (mmHg)	149.7	20.6	144.6	17.3	0.083	147.5	19.0	142.6	18.3	0.098
Diastolic blood pressure (mmHg)	87.2	8.0	88.7	9.7	0.241	84.0	9.3	85.3	9.1	0.376

In health behaviours, three differences were found between the groups (Table 3). Medication for hypertension and diabetes was more common in both genders among those who reported having CHD in the ten-year follow-up than in the others. A corresponding significant difference was found in the use of statins in women. In men there was a trend towards the difference in the use of statins. Men who were ‘heavy drinkers’ seemed to have a diminished risk for developing CHD.

**Table 3:** Health behaviours at baseline and incidence of coronary heart disease in men and women in the ten-year follow-up

	Men						Women					
	Coronary heart disease						Coronary heart disease					
	Yes		No				Yes		No			
	N	%	N	%	Chi-squared	*p*- value	N	%	N	%	Chi-squared	*p*-value
Heavy drinking												
No	53	10.3	461	89.7			43	5.5	733	94.5		
Yes	4	3.3	119	96.7	6.070	0.014	1	4.3	22	95.7	0.061	0.805
Daily smoking												
No	45	8.3	498	91.7			39	5.4	685	94.6		
Yes	12	12.8	82	87.2	1.973	0.160	5	6.7	70	93.3	0.214	0.644
Regular physical exercise												
No	28	8.7	293	91.3			15	4.8	295	95.2		
Yes	29	9.2	287	91.3	0.040	0.841	29	5.9	460	94.1	0.435	0.510
Use of statins												
No	46	8.2	513	91.8			36	4.9	692	95.1		
Yes	11	14.1	67	85.9	2.898	0.089	8	11.3	63	88.7	4.970	0.026
Medication for hypertension												
No	38	7.7	454	92.3			25	4.1	587	95.9		
Yes	19	13.1	126	86.9	3.978	0.046	19	10.2	168	89.8	10.160	0.001
Medication for diabetes												
No	51	8.3	560	91.7			40	5.1	746	94.9		
Yes	6	23.1	20	76.9	6.641	0.010	4	30.8	9	69.2	16.207	<0.001

Finally, we calculated multivariate logistic regression models separately in men and women for the risk for coronary heart disease. We only included the variables that in univariate analyses significantly associated with coronary heart disease or had a trend towards a significant association (*p* < 0.10). Blood glucose and the use of drugs for diabetes were highly correlated with each other, and we chose to use only blood glucose in these analyses. For the same reason, systolic blood pressure was included in the models, and use of drugs for hypertension was excluded.

In men, pessimism associated statistically significantly with the risk for CHD. In women, pessimism did not associate with the risk for CHD (Table 4). To highlight the significance of pessimism as a risk factor for CHD, we compared the highest and the lowest quartiles of pessimism in a similar model. Those men who were in the highest quartile of pessimism had an over four-fold adjusted risk for CHD compared to those in the lowest quartile (adjusted OR 4.11, 95 % CI 1.68–10.04, *p* = 0.002). No difference was found in women between the highest and lowest quartiles of pessimism (adjusted OR 1.56, 95 % CI 0.57–4.29, *p* = 0.386).

**Table 4:** Risk for coronary heart disease in men and women during ten-year follow-up

	Risk for coronary heart disease			
	Men		Women	
	OR	95 % CI	OR	95 % CI
Age (years)	1.03	0.99–1.08	1.08	1.03–1.13
Body mass index (kg/m^2^)	1.00	0.85–1.18	0.99	0.87–1.12
Blood glucose (mmol/L)	1.40	1.09–1.79	1.40	1.11–1.77
Waist circumference (cm)	1.00	0.95–1.06	1.02	0.96–1.07
Systolic blood pressure (mmHg)	1.01	0.99–1.03	1.00	0.99–1.02
Use of statins (yes vs no)	1.55	0.73–3.25	1.67	0.71–3.94
Heavy drinking (yes vs no)	0.32	0.11–0.93	…	…
Pessimism (score)	1.10	1.00–1.22	1.07	0.94–1.20

*OR* fully adjusted odds ratio, *95 % CI* 95 % confidence interval

## Discussion

Our main finding is that pessimism was a clear risk factor for coronary heart disease in men even after adjusting for classical well-known risk factors. Moreover, optimism did not associate with the incidence of CHD and it was not a protective factor. This finding contradicts some previous studies [17–21], which have approached optimism/pessimism as a unidimensional mental construct, whereas our approach was bidimensional.

Our findings highlight the need to scrutinise optimism and pessimism separately as two independent variables. Optimism is not the same as the absence of pessimism and vice versa. According to our findings, the protective factor against CHD is not optimism as previous studies have suggested [17–21]. Rather, our study suggests that the protective factor is a lack of pessimism. This observation would remain unnoticed if optimism and pessimism were seen as part of the same unidimensional construct.

While pessimism seemed to be a clear risk factor for CHD among men, such a connection was not detectable among women. Similar gender differences between psychosocial factors and overall well-being have been noticed in some other studies, too. For example, in a Japanese study of over 88,000 men and women, a low perceived level of life enjoyment was a risk factor for stroke and CHD among men, while among women, the level of life enjoyment was not associated with elevated risks of cardiovascular disease incidence [34]. In another study concentrating on optimism, pessimism and depression, the connection between pessimism and depression was much stronger among men than among women [35]. The mechanism of this gender difference is not fully understood. Differences between men and women in somatic responses to stress may at least partly explain this phenomenon. Cardiovascular reactivity to stressors (e.g. the rise in the levels of blood pressure and heart rate) seems to be more significant among men than among women [36, 37]. In addition, the neuroendocrine response to stress seems to be greater among men than among women. Plasma ACTH and cortisol levels rise more significantly in men than in women in situations of stress, showing that men exhibit greater activation of the hypothalamic–pituitary–adrenal axis to psychological stress [38]. These findings suggest that men have a more remarkable connection between psychosocial factors and CHD than women, as was seen in our study.

When studying optimism and pessimism and their impact on physiological health and CHD in particular, several possible mechanisms have been discovered. Pessimism has been found to associate with inflammation and endothelial dysfunction [39] and shorter telomere length [40] in older men. Optimism is associated with higher carotenoid concentrations and fruit and vegetable consumption as well as a lower smoking rate, which are potential pathways underlying the association [41]. Furthermore, optimism is associated with a healthier diet and a healthy lipid profile, and a lower body mass index may partially explain the association [42] between optimism/pessimism and CHD. Conversely, optimism did not associate with hypertension [43]. In general, optimists have healthier lifestyles [44]. They smoke less, are more physically active, consume more fruit, vegetables and whole-grain bread, and use alcohol in more moderate amounts. Nevertheless, pessimism was established to be a risk factor for CHD in our study, even in logistic regressions including, e.g. smoking and high blood glucose and cholesterol.

According to our study, it is important not only to explore the classical well-known physiological risk factors for CHD when planning the prevention of CHD; it is also important to pay attention to psychosocial components. The degree of pessimism seems to have a substantial effect on the likelihood of developing CHD among men, regardless of the amount of optimism. Measuring pessimism is quite easy and it consumes very little time. Once the amount of pessimism – which seems to be one of the significant risk factors for CHD – is ascertained, it is easier to define who is in the greatest need of preventive actions concerning CHD.

The strength of this study lies in its design. The study was a prospective ten-year follow-up survey based on randomly selected individuals from the older population, with equal numbers of both sexes and representatives of all the age groups invited. The study group can be seen as a comprehensive one. Compared to earlier studies concerning the connection between CHD and optimism and pessimism, in our study life orientation was measured using the complete test pattern of the LOT-R, thus giving more reliable answers.

There are a few limitations in this study as well. One of them is that we could only use self-reports. We did not have access either to the authentic medical files of the population or to the cause-of-death statistics. It is expected that there were more persons with cardiovascular disease among those who died during the follow-up. However, the numbers received by self-reports are quite similar to the incidence rates of CHD that can be calculated from the official statistics for the same-aged population in Finland (8.5 % in men, 3.4 % in women) [45, 46]. Another limitation is that we had relatively small numbers of cases in the study groups due to the separate analyses of men and women. There may have resulted type 2 statistical errors, e.g. it may be possible that we missed some real differences between the groups. Separate analyses for men and women were, however, essential to find out an obvious gender difference in the association between pessimism and the incidence of CHD. Finally, it is probable that poorly functioning and institutionalized persons had a lower participation rate than community-dwelling subjects. It is probable that the incidence of CHD would have been higher in those populations, but it is not known whether there are any differences in pessimism between these groups and the rest of the population.

## Conclusions

Pessimism seems to be quite a significant risk factor for coronary heart disease in men, while optimism does not provide protection. Separating optimism and pessimism improves the prognostic values of the connection between these personality traits and coronary heart disease.
